# Development of a larval fathead minnow optomotor response assay for assessing visual function

**DOI:** 10.1016/j.mex.2020.100971

**Published:** 2020-06-20

**Authors:** Julie C Krzykwa, Marlo K Jeffries

**Affiliations:** Texas Christian University, Department of Biology, 2800 S. University Drive, Fort Worth, TX 76129, United States

**Keywords:** Fathead minnow, Optomotor response assay, Vision, Behavior

## Abstract

The optomotor response (OMR) is a position stabilizing reflex to whole-field visual motion, demonstrated by fish instinctually moving to follow alternating black and white stripes. The OMR assay is used to assess the visual function of fish. While researchers have been using the OMR assay for years to evaluate the visual acuity of fish, no standardized methods exist. Therefore, the goal of the present study was to develop a standardized protocol for the measurement of OMR in larval fathead minnows, a common model species in ecotoxicology. Results indicate that OMR is a potential endpoint for the assessment of vision in larval fathead minnows and can be quantified as early as 9 dpf. When running the OMR assay, a 4-min acclimation period should be implemented. There did not appear to be a learning component to response latency; however, high variation in likely impeded the ability to detect significant differences.

• Optomotor response of fathead minnow larvae can be quantified as early as 9 dpf.
• When running the assay, a 4-min acclimation period should be implemented.
• Due to high variation, response latency is not recommended for use as an endpoint.

## Graphical abstract

**Figure:**
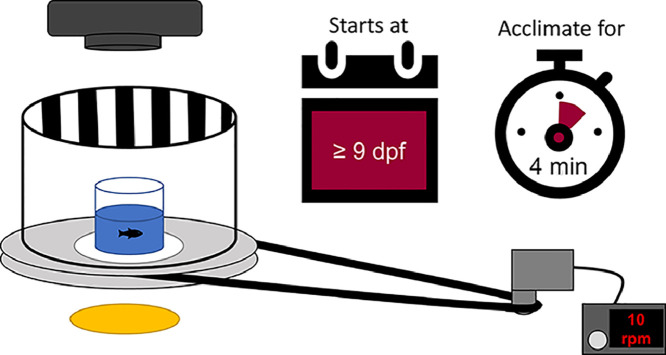

**Table:** Specifications Table

Subject area	Agricultural and Biological Sciences
More specific subject area	Animal behavior
Method name	Optomotor response assay
Name and reference of original method	Rock, Irvin, and Deborah Smith. “The Optomotor Response and Induced Motion of the Self.” Perception 15, no. 4 (August 1986): 497–502. https://doi.org/10/c74ktx.
Resource availability	https://www.ffmpeg.org/ https://imagej.nih.gov/ij/

## Background

The optomotor response (OMR) is a position stabilizing reflex to whole-field visual motion, demonstrated by fish instinctually moving to follow alternating black and white stripes (Rock and Smith 1986). The OMR assay is used to assess the visual function of a fish and has been utilized in ecotoxicology research to identify the impacts of various chemicals on visual acuity and behavior [4], [5], [8]. Typically, the fish is given a set amount of time in the OMR chamber and the amount of time the fish spends swimming in the same direction as the stripes (i.e., following time) is quantified. Decreases in following time are thought to indicate reduced visual acuity [10]. Another metric used for assessing OMR is the measurement of response latency—the amount of time it takes the fish to respond to a change in the direction of the rotating stripes [11]. While researchers have been using the OMR assay for years, no standardized methods exist, which limits the utility of OMR data for the purpose of environmental risk assessment [6,7]. The lack of method standardization is exemplified by the variability in the age of fish used, the amount of time a fish is given to acclimate to the OMR chamber, the amount of time the fish spends performing the assay, and in the approaches utilized to quantify the response [2], [3], [4], [5],8]. The absence of standardization makes it difficult to compare results across multiple studies and could lead to the use of insufficient methods. As such, there is a need to develop standardized OMR methods to address these issues. The goal of the present study was to develop a standardized protocol for the measurement of OMR in larval fathead minnows, a common model species in ecotoxicity testing [1]. Specifically this study sought to determine: 1) the age at which OMR could be quantified in fathead minnows, 2) the amount of acclimation time needed before recording OMR, 3) whether OMR performance was reproducible between different replicates of fish, 4) what depth of water to use in the OMR chamber, 5) whether position of the OMR chamber influenced fish response, and 6) whether response latency is a useful endpoint for the OMR assay.

## Method details

**Fig. 1:**
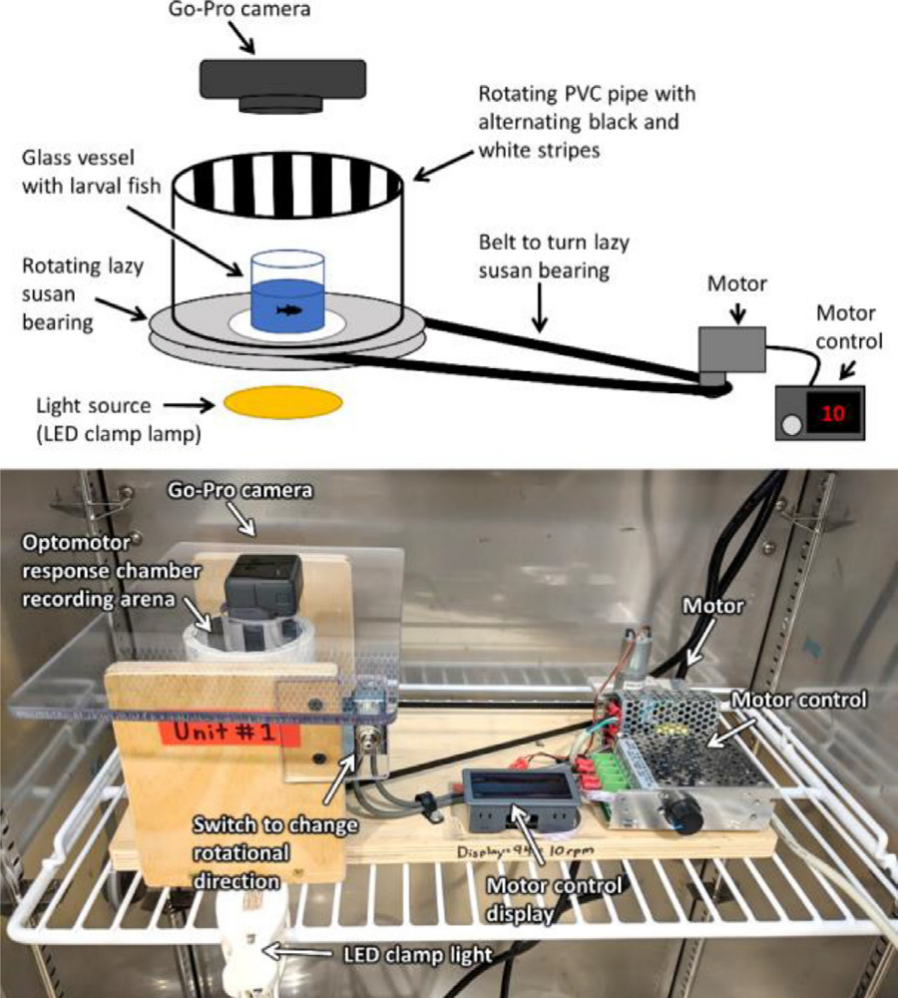
Detailed schematic of the optomotor response chamber, with the different components indicated.

**Fig. 2:**
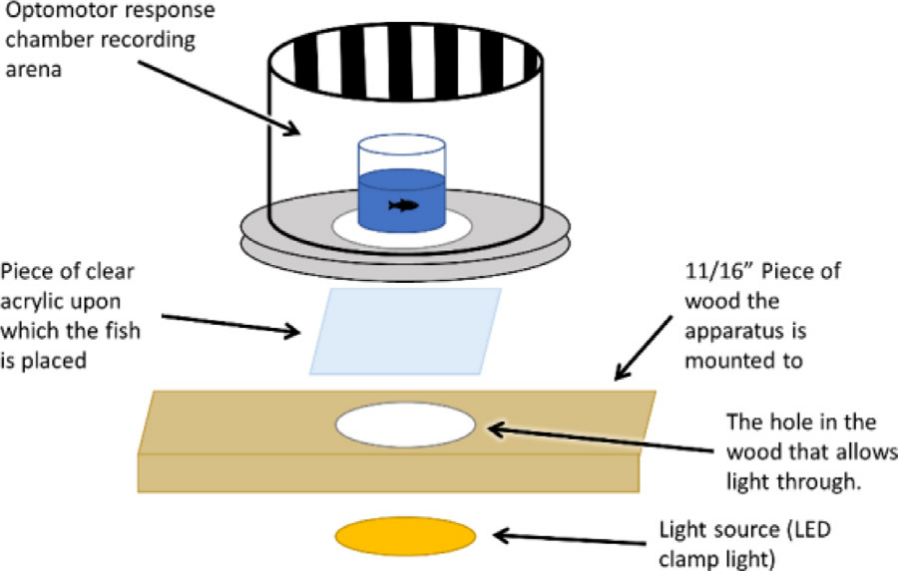
Detailed schematic of the optomotor response chamber recording arena and light source.

On mornings when OMR was measured, fish were given 1 h to feed before the initiation of measurements. Measurements were limited to the number of videos that could be recorded within 6 h of the morning feeding [9]. The OMR chambers were kept in an incubator (Panasonic MIR-254) at 27 °C with the incubator lights turned off during the measurement of OMR. Each chamber consisted of a rotating drum with alternating black and white stripes, a motor to turn the drum, and a GoPro Hero 5 Camera (GoPro, Inc.) mounted above to record the fish behavior (Fig. 1). All components were mounted on plywood that had an opening to allow light to enter the recording arena of the OMR chamber (Fig. 2). Clamp spotlights (IKEA Nävlinge) with LED light sources (warm white, 2700 Kelvin) were used as they emitted no heat and were flexible for positioning. Light levels were dimmed to ~590 lx by placing a piece of white printer paper under the OMR chamber. This ensured that the light level was not so bright that the image of the larval fish in the chamber was washed out in recordings. Fish were placed in a custom-built glass vessel (36 mm wide x 75 mm tall) inside the rotating drum (100 mm tall x 88 mm inner diameter) and the stripes rotated at a speed of 10 rpm. Cards with identifying information were used to keep track of fish and recorded at the beginning of each video to identify the fish in the recording. Individual larvae were transferred to a vessel containing 10 mL of fresh, warmed dechlorinated municipal water and placed in the recording arena of the chamber. Larvae were given 14 min in the chamber before being removed and transferred back to their original container. For the first 9 min, there were no direction changes, with the drum turning in the same direction the whole time, allowing for 4 min of acclimation and 5 min of following time measurement. After the first 9 min, the rotational direction of the drum was altered every minute for 5 min for the measurement of response latency.

All videos were recorded as MP4 video files via a GoPro Camera and then converted to 10 fps TIFF stacks using ffmpeg (https://www.ffmpeg.org/) via a batch file with the following code:

@echo offsetlocal enabledelayedexpansion

for%%f in (*.MP4) do ffmpeg -y -ss 00:04:00 -i "%%f" -r 10 -qscale 0 -vf scale=825:464 "%%~nf_10fps.avi"

for%%f in (*.AVI) do ffmpeg -i "%%f" -pix_fmt rgba -compression_algo deflate%%~nf_%%05dtiff

for%%a in (*.TIFF) do (

set *f*=%%a

set *g*=!f:~0,8!

md "!g!" 2>nul

move "%%a" "!g!"

)

pause

This code only converted the video after the 4 min mark to account for acclimation time. It also moved all the TIFF files into folders named according to the original MP4 so that TIFF stacks were easily navigable and could be tracked back to the original files. Because the first 4 min were not included in the TIFF stack—which included the segment with the fish ID—the video analysis was done blind and the fish ID was later determined from the original MP4 file. To quantify OMR, the TIFF stacks were first imported into ImageJ [12] as virtual stacks. The slice numbers of direction changes by the fish were then recorded in an excel document, with the swimming direction of the fish (following the stripes or opposite of the stripes) also being recorded. For videos in which response latency was measured, the slice number at which the stripes changed direction was recorded and the slice number at which the fish initially changed directions was recorded to determine how long it took the fish to notice the direction change. To account for the 10-fps speed of the videos, the number of slices that the fish spent swimming was divided by 10 to convert to seconds. A detailed protocol for the analysis of OMR videos, and an example excel file, are available as supplemental files. This method of using TIFF stacks in ImageJ allowed for finer resolution of direction changes by the fish. An alternative method of watching the video play at a reduced speed while timing fish direction changes with a stop watch was also attempted and was found to be stressful for the technician quantifying OMR and slower than the TIFF stack method.

## Method development and validation

All procedures involving fathead minnows were conducted per Texas Christian University (TCU) Institutional Animal Care and Use Committee approved methods (Protocols 1707 and 1708). Fathead minnow used in the present study were produced from the TCU fathead minnow colony which was originally sourced from Hydrosphere and Aquatic Research Organisms. Fathead minnow embryos at < 24 h post fertilization were placed in an aerated beaker and kept in an incubation chamber (Panasonic, MIR-254) at 27 °C with a 16 h light: 8 h dark photoperiod until hatch, at which time point larvae were transferred to crystalizing dishes containing 250 mL of dechlorinated municipal water (*n* = 10/dish). Dishes were subject to 80% water changes daily, and larvae were fed 1.06 mg Artemia nauplii/larva twice daily starting at 6 days post fertilization (dpf).

### When do larval fathead minnows begin displaying quantifiable OMR?

**Fig. 3:**
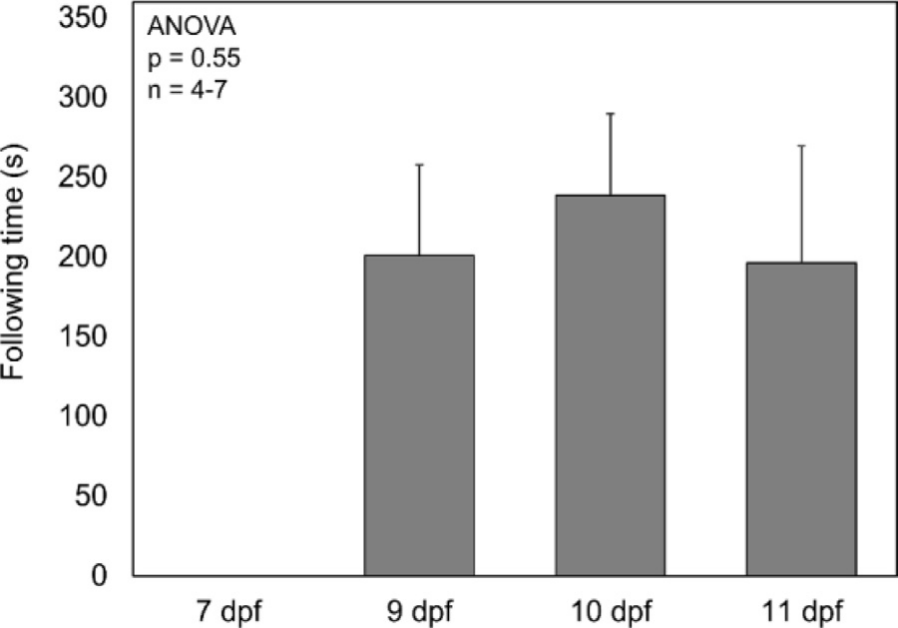
Average following time of larval fathead minnows. Error bars indicate standard deviation. dpf = days post fertilization.

To determine when fathead minnow larvae begin displaying OMR, measurements were made on 7, 9, 10, and 11 dpf. Optomotor response could not be quantified until 9 dpf in fathead minnow larvae. Once the larvae did show a quantifiable response, there were no significant differences in the performance of larvae between days 9, 10 and 11 (Fig 3, ANOVA, *p* = 0.55).

### How long of an acclimation time is needed?

**Fig. 4:**
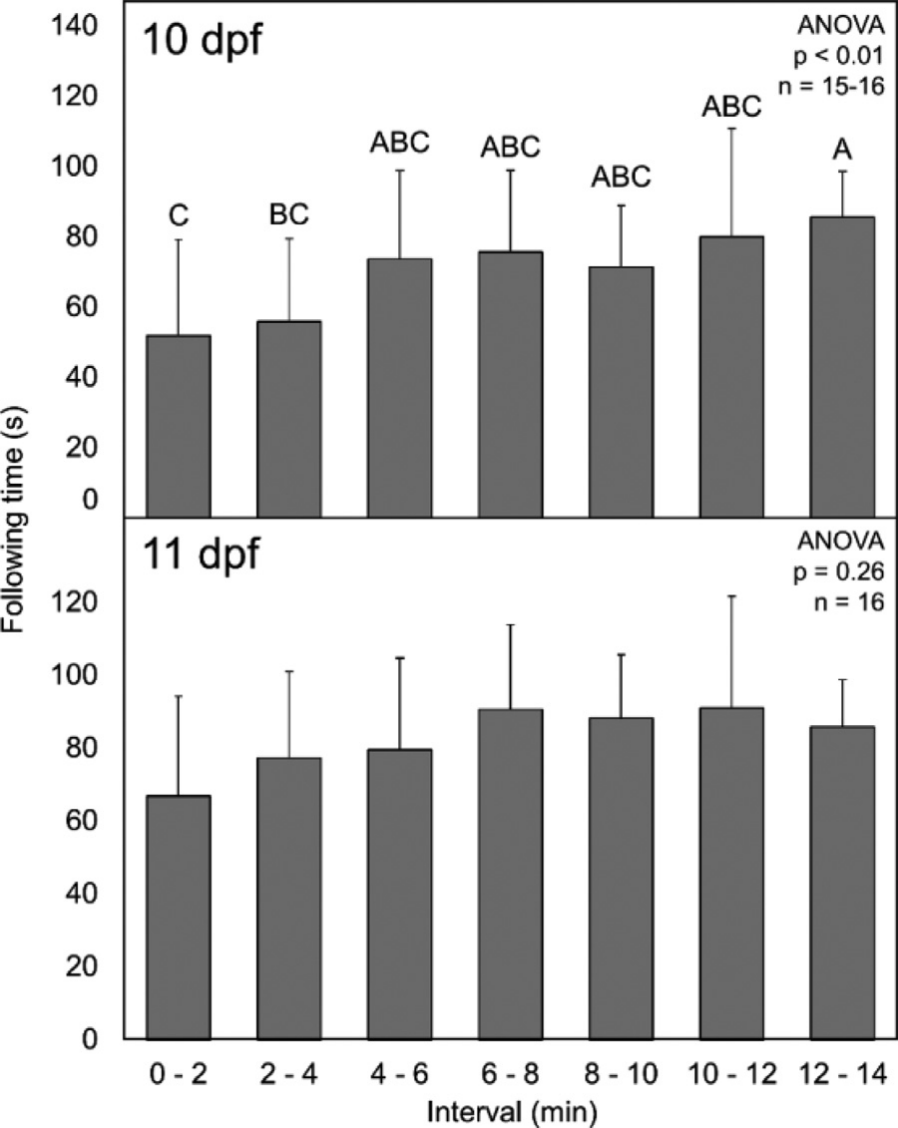
Average following time of larval fathead minnows over 2-min intervals at 10 and 11 days post fertilization (dpf). Error bars indicate standard deviation.

As a result of inconsistent acclimation times reported in the literature, and a desire to use as short of a time as possible to increase the number of fish that could be sampled/day, it was necessary to determine how long fish needed to acclimate to the OMR chamber before quantifying OMR response. To do this, following time was measured in 2 min intervals from the time the larvae was placed in the OMR chamber through 14 min of rotation for a total of seven 2-min intervals. The mean duration of following time was then compared between each interval at 10 and 11 dpf. There were significant differences between intervals at 10 dpf (Fig 4, ANOVA, *p* < 0.01), but not at 11 dpf (Fig 4, ANOVA, *p* = 0.26). At 10 dpf, the mean following time over 0 to 2 min and 2 to 4 min was significantly lower than the mean response time at 12 to 14 min. There were no significant differences in following time between intervals at any age after 4 min. The lack of significant differences between groups after 4 min indicates that at least a 4 min acclimation should be implemented when measuring OMR.

### Are there differences between different replicates?

**Fig. 5:**
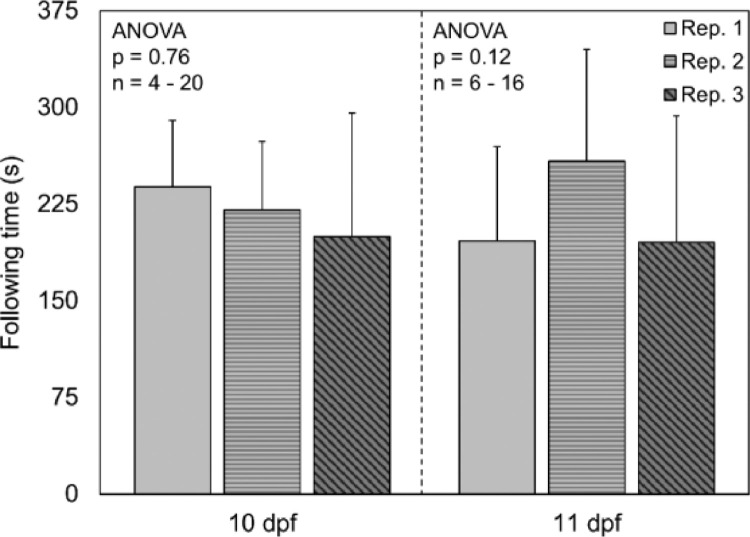
Average following time of larval fathead minnows from different replicates of fish. Error bars indicate standard deviation. dpf = days post fertilization.

The time spent following for three different replicates of fish was also compared at 10 and 11 dpf to make sure OMR was a reproducible response. There were no significant differences in following time between the three different replicates of fish at 10 or 11 dpf (Fig 5, ANOVA, *p* > 0.12). These results show that OMR is reproducible response.

### Does the depth of water in the vessel influence the results?

**Fig. 6:**
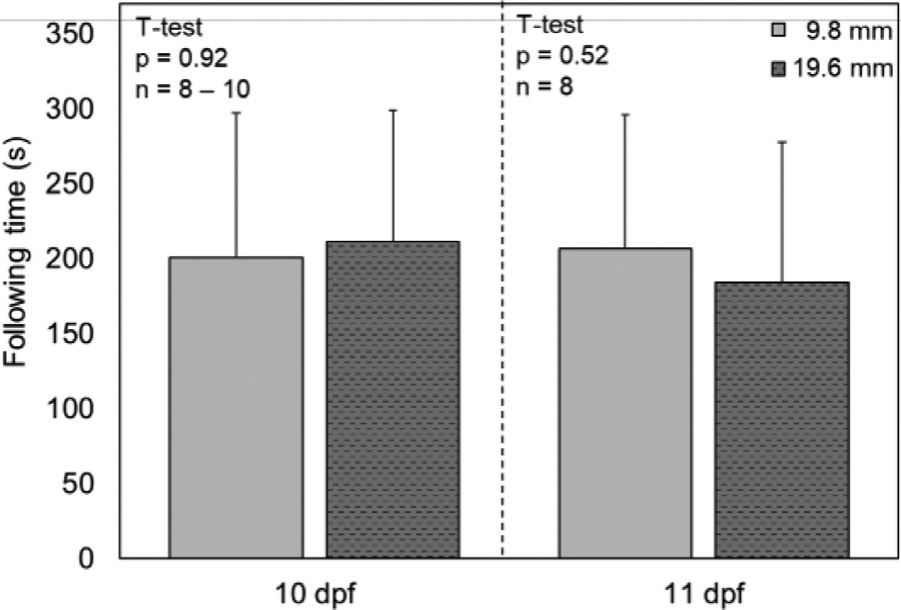
Average following time of larval fathead minnows with different depths of water in the OMR vessel. Depths of 9.8 and 19.6 mm correspond to volumes of 10 and 20 mL, respectively. Error bars indicate standard deviation. dpf = days post fertilization.

To determine if the depth of water in the glass OMR vessel influenced the performance of fish in the OMR assay, two depths were evaluated. Fish were placed into 10 or 20 mL of dechlorinated municipal water in the vessel, which corresponded to depths of ~9.8 and 19.6 mm, respectively. The mean duration of time spent following was compared for the two depths at 10 and 11 dpf. No significant differences were found in the following time between the two depths at either day (Fig 6, T-test, *p* > 0.52). The lack of differences shows that, for at least the depths evaluated, performance of fish in the OMR assay is not impacted by the depth of water in the vessel.

### Does the position of the OMR chamber influence results?

**Fig. 7:**
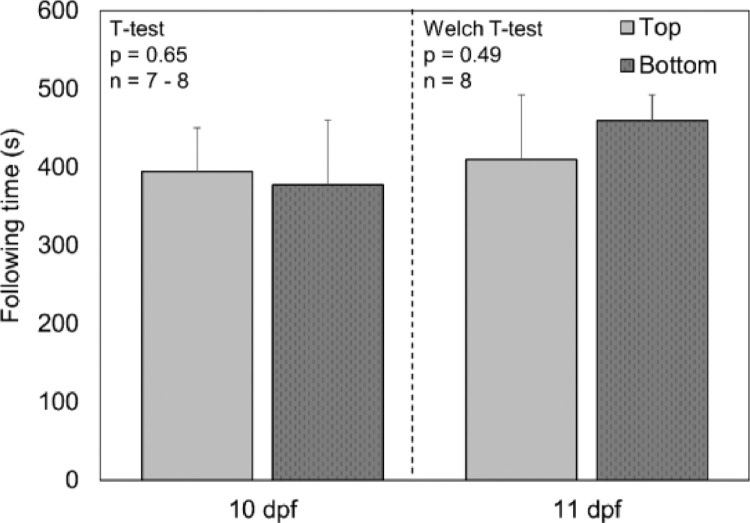
Average following time of larval fathead minnows from optomotor response chamber on the top and bottom shelves of the incubator. Error bars indicate standard deviation. dpf = days post fertilization.

The two OMR chambers being used were on different shelves in the incubator, potentially causing a shelf-effect or a shift in the field of view. The performance of fish in the two OMR chambers was compared to ensure that there was no impact of the OMR chamber and/or where it was located in the incubator. There were no significant differences between the average performance of fish in the OMR chamber on the top shelf and the OMR chamber on the bottom shelf at 10 dpf (Fig 7, *t*-test, *p* = 0.65) or at 11 dpf (Fig 7, Welch *t*-test, *p* = 0.49) indicating that the position of the OMR chamber did not influence the field of view

### Is response latency a useful OMR endpoint?

**Fig. 8:**
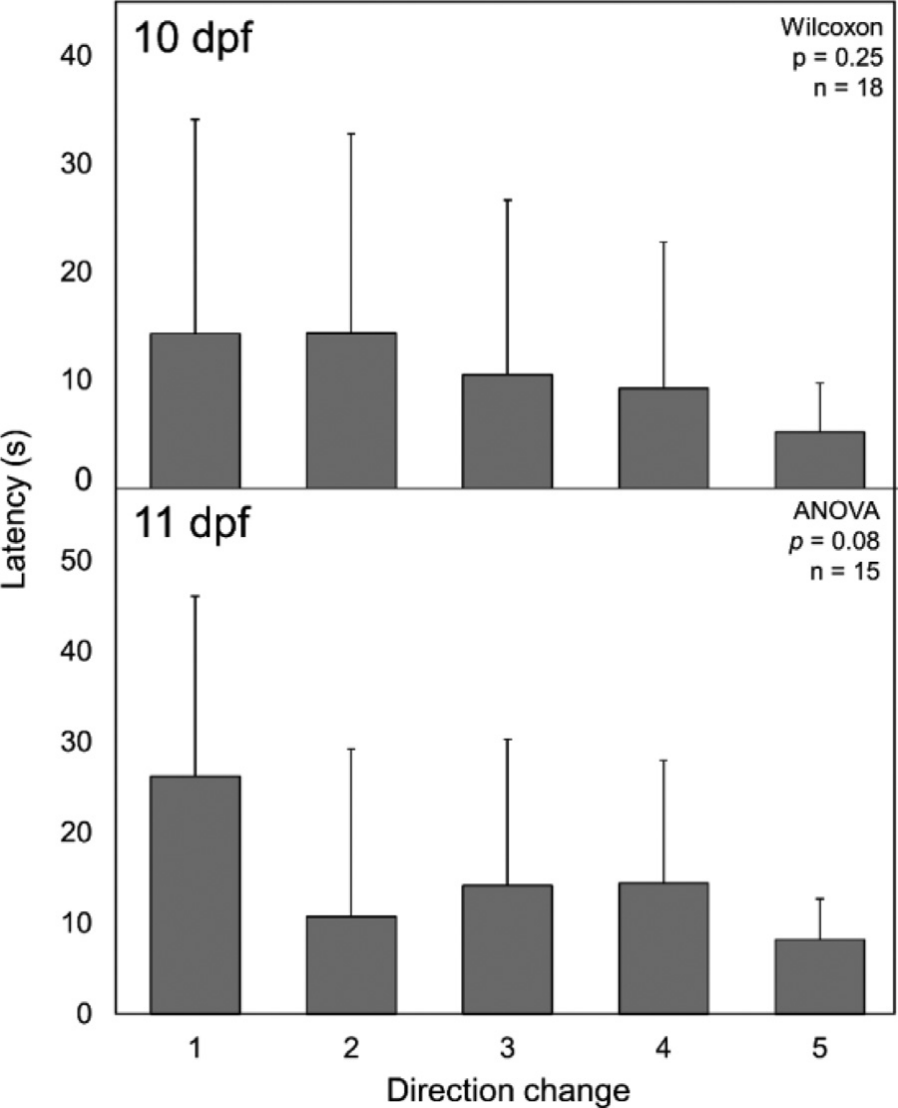
Average response latency of larval fathead minnows over five directional changes. Error bars indicate standard deviation. dpf = days post fertilization.

In addition to developing standardized methods for measuring OMR via following time, the utility of response latency as a secondary OMR endpoint was investigated. Due to concerns of a learning component to response latency, it was measured five times with a 1 min interval between direction changes. The mean time until direction change was compared for each interval at 10 and 11 dpf. There were no significant differences in response latency over the 5 trials on either day (Fig 8, ANOVA or Wilcoxon, *p* > 0.08.)

## Conclusions

Overall, the results of this study suggest that larval age and acclimation time can impact OMR results, while the depth of water in the test vessel and position of the OMR chambers in an incubator are less likely to influence assay outcome. Specifically, this study indicated that larval fathead minnows utilized in OMR assays should be a minimum of 9 dpf and that an acclimation period of at least 4 min should be employed. Though the depth of water in test vessels (in this study 9.8 or 19.6 mm) did not affect OMR performance, deviations from these depths are not recommended without confirmation that they do not impact OMR. There did not appear to be a significant learning component to response latency; however, the high variation in response latency likely impedes the ability to detect significant differences between groups. A post-hoc power analysis (given the variance from the 5th direction change at 11 dpf, α = 0.05 and β = 0.2) revealed that the minimum total sample size needed to detect a 20% change in response latency is approximately 828 fish, well beyond the sample size used in the present study (*n* = 15 – 18). In comparison, a post-hoc power analysis for following time (given the variance from replicate 2 at 10 dpf, α = 0.05 and β = 0.2) revealed that the minimum total sample size needed to detect a 20% change is approximately 14 fish, a more achievable sample size. The need for large sample sizes to detect differences between groups limits the utility of response latency as an endpoint in the OMR assay. This is especially true in cases where there may be additional confounding factors such as genetic differences or differences in the age of the fish being utilized in the assay [13,14]

## Declaration of Competing Interests

None.
